# Effects of School-Based Exergaming on Urban Children’s Physical Activity and Cardiorespiratory Fitness: A Quasi-Experimental Study

**DOI:** 10.3390/ijerph16214080

**Published:** 2019-10-23

**Authors:** Sunyue Ye, Zachary C. Pope, Jung Eun Lee, Zan Gao

**Affiliations:** 1 Institute of Child Development, Jiaxing University, Jiaxing 314000, China; yesunyue@zju.edu.cn; 2 School of Public Health, University of Minnesota, Minneapolis, MN 55455, USA; popex157@umn.edu; 3 Department of Applied Human Sciences, University of Minnesota, Duluth, MN 55812, USA; junelee@d.umn.edu; 4 School of Kinesiology, University of Minnesota, Minneapolis, MN 55455, USA

**Keywords:** accelerometers, active video games, physical activity, sedentary behavior, school intervention

## Abstract

Background: Modern-day technology is appealing to children. Few studies, however, have conducted longitudinal analyses of a school-based exergaming program’s effect on physical activity (PA) behaviors and fitness in children. Therefore, this study examined the longitudinal effect of an 8-month school-based exergaming intervention on children’s objectively-measured PA and cardiorespiratory fitness (CRF). Materials and Methods: Eighty-one fourth grade students (${\bar{X}}_{\mathrm{age}}$ = 9.23 ± 0.62; 39 girls; 54.3% African American, 30.9% Non-Hispanic White, 14.8% other) participated in this study from 2014–2015. The intervention school’s children participated in a once-weekly 50-min exergaming intervention during recess throughout the school year, while the control school continued regular recess. Children’s in-school PA and sedentary behavior (SB) were measured with ActiGraphGT3X+ accelerometers, with CRF assessed via the half-mile run. All measurements were taken at baseline, mid-intervention (four months) and post-intervention (eight months). Repeated-measures two-way ANCOVAs using age and race as covariates were conducted to examine between-school differences over time for SB, light PA (LPA), moderate-to-vigorous PA (MVPA), and CRF. Results: Significant time by group interactions were observed for LPA, *F*(1, 79) = 7.82, η^2^ = 0.09, *p* < 0.01, and MVPA, *F*(1, 79) = 4.58, η^2^ = 0.06, *p* < 0.05, as LPA increased among the control group, while MVPA increased among intervention group. Children in both groups experienced decreased SB during the intervention (intervention: −7.63 min; control: −17.59 min), but demonstrated lower CRF over time (intervention: +46.73 s; control: +61.60 s). Conclusions: Observations suggested that school-based exergaming implementation may be effective in increasing children’s MVPA and decreasing their SB over the course an academic year (i.e., ~eight months). More research is needed, however, to discern how modifications to school-based exergaming might also promote improved CRF in children.

## 1. Introduction

Childhood obesity continues to be a substantial global public health burden [1,2]. This epidemic has been attributed to high levels of physical inactivity and poor dietary behaviors, with underserved minority (e.g., African American) children most susceptible [3]. Indeed, previous studies have indicated that underserved minority children are more likely to be overweight and/or obese than non-Hispanic White children [4,5]. Higher childhood overweight/obesity prevalence is concerning as it places this population at greater risk for developing cardiovascular disease, type 2 diabetes, and other non-communicable chronic diseases in adulthood [6]. Regular physical activity (PA) participation during childhood, however, plays a key role in decreasing children’s future non-communicable chronic disease risk [7]. Thus, the attenuation and prevention of childhood obesity is important and necessitates innovative and effective interventions to promote children’s PA participation and improve their physical fitness—particularly among underserved minority children.

Despite non-exergame video games’ potential negative physiological health impact (e.g., obesity), exergaming has the potential to assist in the promotion of healthier lifestyles among children [8,9]. Exergaming refers to video games requiring partial- or full-body movements to participate in gameplay, with the participant’s body acting as the de facto controller [10]. Over the past decade, exergaming’s technological advances have led to the development of new interactive exercise strategies and have influenced the quality of population-based exergaming PA interventions [11,12,13]. Given these technological advances, school-based exergaming has been increasingly employed as an innovative and fun approach for promoting PA among children [14,15]. Specifically, exergaming capitalizes on children’s interest in computer and video interaction by combining interactive exercise equipment and activities to get children moving and physically active. For example, Dance Dance Revolution (DDR) combines real physical dancing requiring fast-foot movement with energetic music and visuals—game components which serve as an important bridge to capture children’s interest and promote a health-enhancing intensity of PA [16].

Select previous studies have suggested that exergaming is effective in promoting children’s PA and cardiovascular fitness [17,18,19]. For example, Maddison et al. [17] concluded that playing exergaming (EyeToy Knockout [boxing], Homerun [baseball], Groove [dancing upper body], AntiGrav [hover-board], and PlayStation 2 Dance UK [dance pad]) over short time periods (5 min per bout) is similar in intensity to light to moderate traditional physical activities, such as walking, skipping, and jogging in children ages 10–14 years. Further, Graf et al. [18] reported that energy expenditure during exergaming (DDR and Nintendo’s Wii Sports) play is comparable to moderate-intensity walking in children (ages 10–13 years). Importantly, however, these investigations have primarily been conducted in laboratory-based settings which limit these findings’ generalizability to other real-world settings (e.g., school-based exergaming programs). It is also notable that these studies mostly included small samples of Non-Hispanic White children. As previously mentioned, minority children are at greater risk for poorer health outcomes versus non-Hispanic White children [4,5], with school-based exergaming interventions among these underserved populations presenting a potentially feasible method of PA and health promotion. Although Gao et al. [20] reported that a 9-month school-based DDR program improved Latino children’s cardiorespiratory fitness, studies investigating exergaming’s long-term effects on underserved children’s PA and fitness have been scarce. 

This study’s purpose, therefore, was to explore the long-term effects of a school-based exergaming intervention on underserved minority children’s PA behaviors and cardiorespiratory fitness (CRF). It was hypothesized that children receiving the 8-month exergaming intervention would demonstrate greater moderate-to-vigorous PA (MVPA), light PA (LPA), and CRF improvements, as well as greater sedentary behavior (SB) reductions versus control children engaging in usual activities. Study observations may inform researchers and health professionals of whether a school-based exergaming program can effectively improve PA behavior and CRF in addition to what components contribute the strongest influence on this program’s effectiveness—particularly among underserved children from low socioeconomic status (SES) communities.

## 2. Methods

### 2.1. Participants and Research Setting

Eighty-one fourth grade students (${\bar{X}}_{\mathrm{age}}$ = 9.23 ± 0.62; 39 girls) from two urban Minnesota elementary schools participated in this study from 2014–2015. Given the constraints imposed by the school district’s placement of children within specific schools and classrooms, we employed a longitudinal quasi-experimental repeated-measures design which used school as the unit of randomization, and randomly selected one school to be the intervention school and the other the control school. Both participating schools had Title-I designations meaning ≥ 50% of children received free or reduced-price breakfast and lunch [21]. Most children within the two schools were African American (54.3%), followed by non-Hispanic White (30.9%), Hispanic American (4.9%), Asian American (4.9%), Native American (1.2%), and Other (3.7%). Full demographic characteristics are available in Table 1. Prior to study initiation, University Institutional Review Board and school district approval were obtained, with informed parental consent and child assent before data collection. Further, all procedures performed with participants were in accordance with the standards of the Institution and/or national research committee and with the 1964 Helsinki declaration and its later amendments or comparable ethical standards [22].

### 2.2. Measures

*Height, Weight, and Body Mass Index (BMI).* Height was measured to the nearest half-centimeter using a Seca stadiometer (Seca; Hamburg, Germany), while weight was measured to the nearest tenth of a kilogram using a Detecto weight scale (Detecto, Webb City, MO, USA). Body mass index (BMI) calculations were completed by dividing weight in kilograms by height in meters squared. These calculated values were then converted to normative percentiles based upon the child’s sex and age, with the Centers for Disease Control and Prevention’s BMI-for-age growth charts used in this conversion [23]. 

*Physical Activity*. Children’s MVPA, LPA, and SB durations were assessed using ActiGraph GT3X+ accelerometers during all school hours. Accelerometers are small devices that measure activity counts by analyzing three-dimensional acceleration data of an individual’s PA. ActiGraph accelerometers have been observed valid and reliable for PA assessment among children [24], with prior literature using these devices to assess PA behaviors among children during exergaming [25]. Accelerometers were positioned over children’s right hip at the level of the superior iliac crest by trained research assistants using an elastic belt. Notably, the following empirically-based cut points (in counts/minute) were used to analyze these PA data: MVPA: ≥2296, LPA: 101–2295, and SB: 0–100 [24]. Per standardized guidelines, children wore the accelerometers for three days at baseline, mid-intervention (four months), and post-intervention (eight months), with follow-up procedures used with any child missing a day to ensure the greatest amount of valid and complete PA data. All PA data was validated within ActiLife (v. 6.13; ActiGraph Corp, Pensacola, FL, USA) and truncated to match the children’s school day (6 h 20 min).

*Cardiorespiratory Fitness.* Children’s half-mile run (HMR) times were used as an indicator of CRF in the present study given its feasibility with a large population in real-world settings. Research assistants measured out the HMR within each school’s gym to avoid confounding factors, due to weather and terrain. Multiple research assistants then timed children in a 1:1 ratio as the children completed the HMR. The HMR has been used as a measure of CRF previously [26]. Akin to the PA assessments, this assessment was completed at baseline, mid-intervention, and post-intervention.

### 2.3. Procedures

Children were individually fitted with ActiGraph accelerometers upon arrival to school by trained research assistants. Next, following classroom teacher approval, children were removed one-by-one from class for height and weight measurements—completed in a separate, private room. These measurements took less than two minutes and were conducted during children’s reading time so as to not conflict with any class lessons. The CRF was completed during the children’s physical education class, with research assistants measuring the HMR course prior to children’s arrival to the gym. Children completed the CRF in groups of 6–8 to ensure that children’s CRF could be tracked by research assistants in a 1:1 ratio; thus, ensuring accurate timing.

Children in the intervention school participated in a once-weekly 50-min exergaming intervention during recess throughout the school year, while the children in control school continued regular recess activities (e.g., non-structure play at outside playground or classroom, gym depending on weather). In detail, within the intervention school, nine exergaming stations were set up within a large classroom, with each station equipped with an Xbox 360 (Microsoft, Redmond, WA, USA) or Nintendo Wii (Nintendo, Kyoto, Japan). Games included, but were not limited to: Just Dance, Wii Fit, Gold’s Gym Cardio Workout, and Kinect Sports. Each station accommodated the gameplay of two children simultaneously, with children rotating stations every 10 min throughout the session. This transition took less than one minute and ensured that children could play most games during each session, while also keeping the children’s interest level in gameplay high. These sessions were initially run by research assistants after which a school staff member began implementing the program autonomously, given the exergaming program’s low-burden nature. All other PA opportunities offered for children at both intervention and control schools were comparable, with no other PA-related programs offered before or after school.

### 2.4. Statistical Analysis

Frequencies and means/standard deviations of children’s demographic characteristics and outcome variables were first calculated for baseline, mid-intervention, and post-intervention. Missing data were imputed by the expectation maximization method [27]. Baseline differences between the intervention and comparison group for all outcome variables were calculated, with Student’s *t*-test employed for continuous variables and Chi-square tests used for categorical variables. Repeated-measures two-way ANCOVAs using age and race as covariates were conducted to examine differences in SB, LPA, MVPA, and CRF between the two schools over time (between-subjects factor: Group; within-subjects factor: Time). All statistical analyses were conducted using SPSS 20.0 (IBM Corp., Armonk, NY, USA), and the significance level was set at 0.05 for all statistical analyses.

## 3. Results

The final analysis included 81 children. Baseline comparisons revealed the control group to have significantly lower age, height and number of African American students versus the intervention group (Table 1). Further, the control group had significantly greater SB, but significantly lower MVPA and CRF compared to the intervention group (Table 2).

Significant time by group interactions were observed for LPA, *F*(1, 79) = 7.82, η^2^ = 0.09, *p* < 0.01, and MVPA, *F*(1, 79) = 4.58, η^2^ = 0.06, *p* < 0.05 (see Table 2). Specifically, intervention children increased MVPA over time (+12.70 min), while the control children only experienced a slight improvement (+2.25 min). The inverse was observed for LPA as control children demonstrated an increase over time (+15.32 min), while the intervention children had a small 5.07-min decrease. Whereas, no significant time by group interaction effects were observed for SB or CRF (*p* > 0.05). Briefly, both groups experienced decreased SB during the intervention (intervention: −7.63 min; control: −17.59 min), but had lower CRF over time (intervention: +46.73 s; control: +61.60 s)

## 4. Discussion

The present study observed that school-based exergaming implementation could result in increased MVPA over the course of one academic year (~eight months). This is a notable observation as research has suggested exergaming programs have resulted in greater increases in school day MVPA relative to usual recess activities in children [28,29,30]. These observations may suggest to researchers and health professionals that a school-based exergaming program might be a feasible manner by which to promote improved PA among underserved minority children.

Previous studies have supported our observations regarding the positive impact of exergaming interventions on children’s light to moderate PA levels [31,32,33]. This improvement may be attributed to improved intrinsic motivation given children’s previously observed enjoyment of exergaming [11]; although previous studies could not discern whether children’s enjoyment could be sustained over time [34]. Our study suggested that a school-based exergaming program offering several stations and game options could sustain children’s PA motivation and subsequently maintain or increase PA over an entire academic year. These observations also indicated that exergaming could be a viable way to enhance PA levels in *minority children* with fewer PA opportunities. This observation mirrors that of a 6-week exergaming intervention wherein Flynn et al. [35] also reported that exergames could be a feasible manner by which to increase PA in minority children who live in poverty-impacted neighborhoods. As mentioned earlier, underserved minority children are more likely than white children to be physically inactive and develop obesity and other chronic diseases in adulthood [3,4,5,6]. Therefore, exergaming, a relatively low-cost PA tool and strategy, could be a viable option for motivating and promoting PA in minority youth. More research needs to replicate these observations, however.

Interestingly, observations suggested that children’s LPA time may be replaced by MVPA over the course of this school-based exergaming intervention. It is possible that the current exergaming intervention motivated children to progress from an initially lower PA intensity (LPA) to a higher PA intensity (MVPA) as the intervention progressed given that 12 exergaming stations offering different games appeared to sustain PA motivation and interest [31]. However, reasons for the elevated SB at mid-intervention among both groups are unclear. One possible explanation is that these variations may be because of seasonal fluctuations in PA, due to weather patterns (e.g., fewer opportunities for PA participation—particularly outdoor PA participation—during the cold winter months). Indeed, Belanger et al. [36] and Hjorth et al. [37] have shown that PA declines and SB increases during winter months. As the current study was completed in Minnesota where winter temperatures have a marked impact on children’s outdoor recess opportunities, this explanation has some plausibility.

No consistent trends were observed regarding exergaming’s effect on CRF in the current study, nor in previous literature. In a previous review, Kari [19] concluded that current evidence does not support the ability of exergaming to promote CRF, despite exergaming’s enjoyable nature and ability to promote light-to-moderate PA. LeBlanc et al. [38] indicated that exergaming’s typical exertion level is not sufficient to meet the guidelines for children of 60 min/day of MVPA. Although a recent study indicated that minority youth (aged 10 to 15 years; 50% overweight or obese) reported high exergaming enjoyment levels and perceived exergames as a manner by which to increase their CRF [35], studies have concluded that light-to-moderate PA participation may not be adequate to improve children’s CRF and that children’s effective PA dose (i.e., PA intensity x PA duration) may be much higher than adults [19,39,40]. Thus, ways to promote more vigorous-intensity exergame gameplay several times per week—while maintaining children’s exergaming enjoyment—may be the focus of future studies of school-based exergaming programs. Specifically, as minority children perceive exergaming as an acceptable means to improve their fitness and PA, they might receive a larger benefit if more exergaming sessions could be offered at school-settings. For instance, exergaming sessions may be offered not only during recess, but also before and after school or even during physical education using culturally-tailored games. As these children are persistently exposed to enjoyable and fun PA environment, the attitude and motivation to participate in PA may be transferred to other PA modalities. However, it should be noted that the indoor nature of exergaming may potentially interfere with outdoor or semi-structured play time. Therefore, it would be wise to utilize exergaming during adverse weather conditions or when there is a shortage of personnel and physical resources to allow children to engage in unstructured outdoor activity.

Major study strengths include: (1) Long-term intervention period (~8-month academic year); (2) use of multiple exergaming stations with a variety of games; (3) the sample of underserved minority children; and (4) PA assessment using accelerometers. However, this study does have limitations. First, randomization occurred at the school-level, not the individual-level. Although school constraints prohibited randomization at the individual-level, future studies might consider individual-level randomization, if possible within the school district. Second, children’s out-of-school PA was not measured in our study. Of note, differing PA levels before and/or after school may have contributed to the variation in children’s CRF; although a recent study reported that children’ performance on Progressive Aerobic Cardiovascular Endurance Run tests was not significantly associated with participation in non-organized PA outside of school [41]. Finally, while the HMR is a valid test of children’s CRF in the field-based settings, the test results may have been influenced by children’s motivation levels to perform the HMR. Therefore, more reliable measures (e.g., cardiorespiratory coordination) that well reflect cardiorespiratory variations are encouraged in future studies [42,43,44].

## 5. Conclusions

The long-term implementation of school-based exergaming could result in increased MVPA and decreased SB in minority children. These results add to the paucity of literature investigating the long-term effects of a school-based exergaming program on children’s PA and fitness. However, the potential negative effects of exergaming interfering with unstructured outdoor activity should also be kept in mind when seeking to implement a school-based exergaming program in underserved schools. Finally, more studies employing similar rigorous designs are needed to explore what modifications might be made to school-based exergaming programs to assist in promoting CRF among children [45]. 

## Figures and Tables

**Table 1:** Baseline demographic characteristics of participants.

Variables	Intervention (*n* = 36)	Control (*n* = 45)	p Value *
Age, years	9.42 (0.77)	9.09 (0.42)	0.017
Gender			0.551
Girls, n (%)	16 (44.4)	23 (51.1)	
Boys, n (%)	20 (55.6)	22 (48.9)	
Race/ethnicity			0.000
African American, n (%)	30 (83.3)	14 (31.1)	
Non-Hispanic American, n (%)	3 (8.3)	22 (48.9)	
Others, n (%)	3 (8.4)	9 (20.0)	
Height, cm	141.69 (6.84)	136.09 (7.53)	0.001
Weight, kg	39.72 (9.15)	37.02 (10.21)	0.219
BMI, kg/m^2^	19.62 (3.52)	19.78 (4.21)	0.856
BMI (percentiles), %	70.00 (28.33)	69.32 (28.16)	0.848

*: Student’s *t*-test for continuous variables and Chi-square test for categorical variables.

**Table 2:** Baseline, mid- and post-intervention physical activity and fitness descriptive and ANCOVAs using age and race as a covariate with repeated measures.

Groups		Physical Activity and Fitness			
		SB Time, min/d	LPA Time, min/d	MVPA Time, min/d	CRF, Seconds
Intervention group	Baseline	796.44 (80.08)	266.85 (63.46)	76.71 (21.95)	368.78 (64.62)
	Mid-int.	815.59 (76.28)	249.41 (52.74)	74.99 (28.78)	393.62 (58.08)
	Post-int.	788.81 (73.07)	261.78 (49.16)	89.41 (29.43)	415.51 (113.83)
Control group	Baseline	826.86 * (56.93)	251.32 (46.85)	61.83 ^†^ (17.23)	325.11 * (85.00)
	Mid-int.	832.73 (68.53)	258.57 (57.57)	48.69 (15.14)	371.60 (71.00)
	Post-int.	809.27 (64.05)	266.64 (51.60)	64.08 (18.00)	386.71 (97.01)
F		1.98	7.82	4.58	0.89
p		0.14	0.001	0.012	0.412
η^2^		0.03	0.09	0.06	0.01

SB, sedentary behavior; LPA, light physical activity; MVPA, moderate or vigorous physical activity; CRF, cardiorespiratory fitness; PA, physical activity. *: *p* < 0.05; ^†^: *p* < 0.01; Student’s *t*-test was employed between intervention group and control group at baseline.

## Data Availability

The datasets used and/or analyzed during the current study are available from the corresponding author on reasonable request.
